# Melanoma-derived cytokines and extracellular vesicles are interlinked with macrophage immunosuppression

**DOI:** 10.3389/fmolb.2024.1522717

**Published:** 2025-01-22

**Authors:** Shankar Suman, Wendy K. Nevala, Alexey A. Leontovich, James W. Jakub, Liyi Geng, Sarah A. McLaughlin, Svetomir N. Markovic

**Affiliations:** ^1^ Department of Oncology, Mayo Clinic, Rochester, MN, United States; ^2^ Department of Quantitative Health Sciences, Mayo Clinic, Rochester, MN, United States; ^3^ Department of Surgery, Mayo Clinic, Jacksonville, FL, United States

**Keywords:** tumor-promoting macrophage, cytokines, extracellular vesicles, melanoma, immunosuppression, tumor microenvironment

## Abstract

Cytokines play a crucial role in mediating cell communication within the tumor microenvironment (TME). Tumor-associated macrophages are particularly influential in the regulation of immunosuppressive cytokines, thereby supporting tumor metastasis. The upregulation of Th2 cytokines in cancer cells is recognized for its involvement in suppressing anticancer immunity. However, the association between these cytokines and tumor-secreted extracellular vesicles (EVs) remains poorly understood. Therefore, our objective was to investigate the connection between tumor-promoting macrophages and melanoma-derived EVs. The analysis from altered cytokine profile data showed that melanoma-derived EVs upregulate Th2 cytokine expression in naïve macrophages, thereby contributing to the promotion of tumor-supporting functions. Notably, many of these cytokines were also found to be upregulated in metastatic melanoma patients (n = 30) compared to healthy controls (n = 33). Overall, our findings suggest a strong connection between melanoma secretory EVs and the induction of tumor-associated macrophages that facilitates the development of an immunosuppressive TME, supporting melanoma metastasis through regulation at both local and systemic levels.

## 1 Introduction

The current understanding of the immune landscape in cutaneous melanoma depicts it as an immunogenic malignancy (Nirmal et al., 2022). Melanoma cells express a plethora of cytokines and growth factors crucial in primary tumor compared to distant metastases (Elias et al., 2010). These melanoma mediators are involved in autocrine and paracrine signaling to maintain the tumor microenvironment (TME) (Suman and Markovic, 2023; Buckels et al., 2019). These mediators which include cytokines and other growth factors as well as membrane bound vesicles, induce the development of immunosuppressive macrophages associated with tumor metastasis through the formation of premetastatic niches (Suman and Markovic, 2023; Salmi et al., 2019; Suman et al., 2024). Tumor-promoting macrophages (TPMs) also known as tumor associated macrophages in melanoma correlate with an unfavorable prognosis and are more prevalent in invasive melanomas than in benign nevi (Salmi et al., 2019). Within the domain of macrophage physiology, a portfolio of cytokines is considered to mediate the fate of a spectrum of functional forms of these cells (Arango Duque and Descoteaux, 2014). Tumor-secreted mediators including tumor-derived extracellular vesicles serve as a stimulus for macrophages in developing immunosuppressive phenotypes (Tian et al., 2023).

Extracellular vesicles (EVs) play significant roles in normal development and various physiological processes by facilitating communication between cells (De Toro et al., 2015). Furthermore, EVs exhibit a range of immunoregulatory functions as the proteins contained within these vesicles are likely involved in essential processes such as vesicle formation and trafficking, signal transduction, cytoskeletal organization, and antigen presentation (Robbins and Morelli, 2014). Tumor-derived EVs are important in conditioning the TME and promoting immune escape through transfer of carcinogenic lipids, nucleic acid, and proteins (Tan et al., 2022). Tumor-derived EVs promote immune evasion and interfere with immune responses in the TME. This process involves various mechanisms, which include induction of apoptosis in CD8^+^ T cells, generation of regulatory T cells (Tregs), suppression of natural killer (NK) cells, inhibition of the maturation and differentiation of monocytes, and enhancement of the suppressive functions of myeloid-derived suppressor cells (MDSCs) (Xie et al., 2022; Wang and Zhang, 2021). Tumor-derived EVs alter the myeloid differentiation pathway, directing toward the MDSC (CD11b+ Gr1+) lineage in a mouse model (Xiang et al., 2009). These tumor derived-EVs further involve in the metabolic reprogramming in macrophages to suppress their function and help developing premetastatic niche (Kersten et al., 2023). Melanoma-derived EVs also play a key role in developing an immune-tolerant microenvironment (Suman and Markovic, 2023) as it increases the expression of programmed cell death ligand 1 (PD-L1) on immature myeloid cells, which suppress T-cell activation (Fleming et al., 2019). Hood et al. showed that melanoma-derived EVs prepare microanatomic niches to facilitate lymphatic metastasis (Hood et al., 2011). Melanoma-derived EVs efficiently spread through the lymphatic system and preferentially bind to macrophages located in the subcapsular sinus (SCS) of tumor-draining lymph nodes (Pucci et al., 2016). When the SCS macrophage barrier is disrupted, tumor-derived EVs can enter the lymph node cortex, interact with B cells, and promote tumor-supporting humoral immunity (Pucci et al., 2016).

Macrophages are commonly classified as M1 and M2 based on their respective proinflammatory and tissue repair or anti-inflammatory functions. Nonetheless, TPMs demonstrate a diverse range of characteristics, encompassing both M1 and M2 phenotypes in the TME (Gao et al., 2022). TPMs are a crucial part of the TME, which play a significant role in various processes, including angiogenesis, extracellular matrix remodeling, immunosuppression, and resistance to therapy (Mantovani et al., 2022). However, the activated form of macrophages acts against tumors by engaging bidirectional interactions with both innate and adaptive immunity (Mantovani et al., 2022). Moreover, M2-like TPM is an essential component that contributes to lymphangiogenesis, immunosuppression, and drug resistance, making them important target for successful immunotherapy (Gao et al., 2022; Wang et al., 2024). The development of immunosuppressive macrophages represents a pivotal aspect of these processes. Soluble factors such as cytokines facilitate communication between tumors and macrophages to maintain the TME and establish a premetastatic niche (Suman and Markovic, 2023). However, their association with melanoma-derived EVs is not fully understood. This study investigates the link between melanoma-derived EVs and the development of TPMs through comprehensive cytokine profiling. Furthermore, it compares these associations in the blood plasma of patients with metastatic melanoma, elucidating the mechanisms by which melanoma cells regulate metastasis through cytokine-mediated pathways.

## 2 Materials and methods

### 2.1 Cell lines, melanoma-derived EV collections, and human macrophage cytokine arrays

**FIGURE 1:**
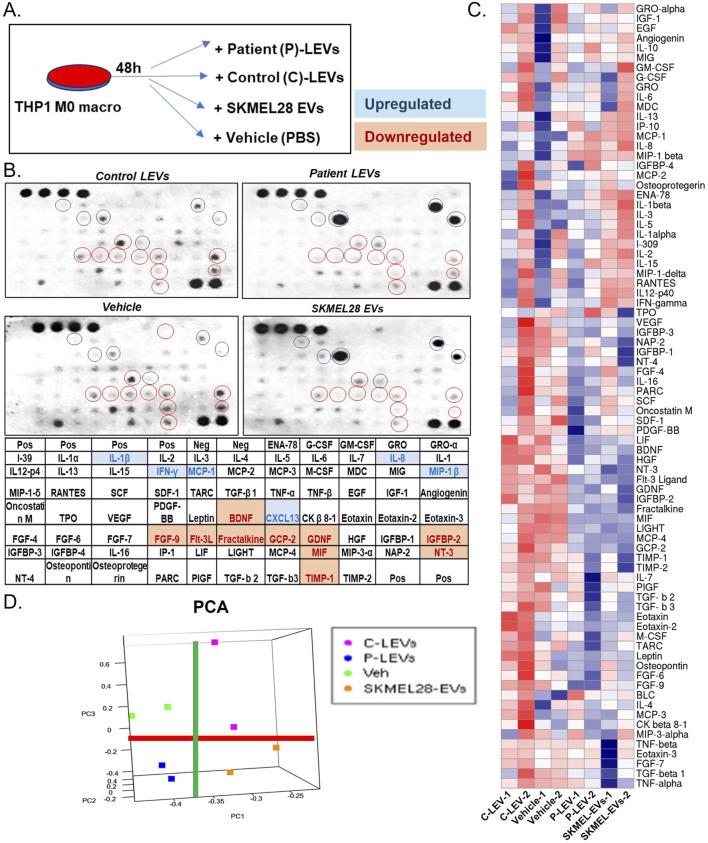
Melanoma-derived EVs transform naïve macrophages through an altered cytokine profile **(A)** Scheme of EV treatment (vehicle, SKMEL28 EVs, control LEVs, and melanoma patient LEVs) to the THP1 M0 macrophages; **(B)** human cytokine assay analysis of naïve macrophages treated with melanoma-derived EVs indicated several altered levels of cytokines, which appear similar in melanoma LEVs and SKMEL28 EVs compared to control LEVs and vehicle groups; **(C)** The heatmap shows all cytokines with altered levels induced by melanoma-derived EVs; and **(D)** principal component analysis (PCA) indicates the separation of melanoma EV-treated groups from control. The red line separates all control (C-LEVs and Veh) from melanoma-derived EVs (P-LEVs and SKMEL28-EVs), whereas the green line separates controls and melanoma-derived EVs.

The human melanoma cell line (SKMEL-28) and monocyte cell line (THP1) were obtained from ATCC, United States, and cells were cultured in the recommended cell culture medium, following given instructions. EVs from SKMEL28 and human lymphatics from control (C-LEVs) and melanoma patients (P-LEVs) were collected by using previously described methods (Maus et al., 2019; Suman et al., 2024). In brief, we extracted LEVs from the lymphatic fluid of patients with melanoma downstream of primary cutaneous melanomas and compared it with those of control individuals (non-malignant post-operative fluid collected from the lymph node dissection field) (Supplementary Figure S1). We performed human cytokine antibody array analysis (RayBiotech, Norcross, GA) by following the manufacturer’s protocol to evaluate the expression levels of 80 human cytokines by the effect of melanoma-derived EVs on naive macrophages (M0) (Figure 1; Supplementary Figure S2). In brief, M0 macrophages were specifically generated from THP1 cells by treating them with 20 ng/mL phorbol 12-myristate-13-acetate (PMA) for 48 h. The adherent M0 cells were washed twice with 1× PBS to remove any residual PMA and non-adherent monocytes. These adherent M0 cells were then treated with EVs in complete Roswell Park Memorial Institute (RPMI) medium for 48 h. After the incubation, cells were washed again with cold 1× PBS to eliminate any remaining media and EVs before preparing the protein lysate for cytokine array analysis. An equal amount of protein from each sample set was applied to the cytokine array membrane according to the manufacturer’s instructions for cytokine analysis. The signals of the spots on the membrane were detected using chemiluminescence methods on the X-ray film. The intensity of protein signals was quantified by densitometric analysis using Quantity One software (Bio-Rad Laboratories, Hercules, CA). After background subtraction, results were expressed as the percentage of the mean of the relative positive controls.

### 2.2 Measurement of plasma cytokine levels

Soluble proteins in plasma are measured using the 38-plex magnetic bead Luminex kit, following the manufacturer’s instructions (Millipore, Burlington, MA). In brief, 25 µL of varying-sized magnetic beads containing antibodies specific to the measured proteins was added to a 96-well plate, with 25 µL of the sample, assay buffer (background control), or standard curve dilutions (3.2–10,000 pg/mL). The beads and samples were incubated overnight at 4°C with gentle shaking. The next day, the magnetic beads were washed three times with wash buffer, and 25 µL of HRP-conjugated secondary antibodies was added and incubated at 4°C for 1 h with gentle shaking. After bead incubation with detection antibodies, 25 µL of streptavidin–PE was added for 20 min. After washing three times, the magnetic beads were enumerated using the Luminex 200 (Millipore, Burlington, MA). The concentrations of proteins were calculated from the standard curve using Milliplex Analyst software (Millipore, Burlington, MA).

### 2.3 Flow cytometry of human blood-derived macrophages

We analyzed the effect of SKMEL28 EVs on human peripheral blood mononuclear cells (PBMCs) derived macrophages from 10 healthy human blood donors. In brief, PBMCs were cultured in RPMI medium for 24–48 h before treating with 5 ng/mL GM-CSF for 3 days. Adherent cells were further treated with control and melanoma-derived EVs for 48 h. Cell surface staining (CD206, CD163, and HLA-DR) and intracellular staining (MCP1, CXCL13, IL8, and MIP-1β) were performed following the manufacturer’s instructions (provided in the supplementary materials, Supplementary Table S1). Flow data were acquired by Bio-Rad ZE5 flow cytometry and analyzed using Flow Jo software v10.0 (Ashland, Oregon).

### 2.4 Bioinformatics and statistical analysis

Normal, tumor, and metastatic tissue gene expressions were analyzed using the TNM plot data server (tnmplot.com) (Bartha and Győrffy, 2021). RNA-seq data were exported from the human skin cutaneous melanoma (SKCM) dataset for IP-10, GCSF, CXCL13, CCL2, CD163, and CD209 and analyzed using normal tissue from the non-cancer patients (n = 474), tumor tissue (n = 103), and metastatic melanoma tissue (n = 368). Furthermore, the gene chip database was used for the analysis of the genes that are absent in the RNA-seq data, which included CCL4, consisting of normal (n = 174), tumor (n = 253), and metastasis (n = 76) tissue genes. The correlation analysis of CD163 with macrophage-associated cytokine genes and other transcription factors was performed in the SKCM dataset using the TIMER2.0 webserver (http://timer.comp-genomics.org/timer/). Principal component analysis and maps as well as hierarchical clustering and heat maps were created using the R programming language (R4.2.2). All the other statistical analyses were conducted using GraphPad Prism V.10.3 software (La Jolla, California, United States).

## 3 Results and discussion

### 3.1 Melanoma-derived EVs transform naïve macrophage phenotypes toward tumor-supportive functions

To evaluate the cellular cytokine changes in the macrophages by the effect of melanoma-derived EVs, we treated THP1 monocytic cells with PMA to induce them to naïve macrophage. Furthermore, to check the cytokine modulation of naïve macrophages by the effect of melanoma-derived EVs, we performed cytokine array analyses of 80 key factors to determine macrophage phenotype state. A control set of EVs was collected from the lymphatic channel (control LEVs) from non-cancerous nodes to compare with the effect of EVs from melanoma-associated lymphatic channels (patient LEVs). We also analyzed SKMEL28 EVs and vehicle control to compare the direct effect of cancer cell EVs. Array data apparently show similar results on cytokine arrays for SKMEL28 and melanoma patient LEVs compared to respective controls (Figure 1). Among these, IL-1β, IL-8, IL-13, MIP-1β, MCP1, and CXCL13 were key upregulated cytokines, and MIF, IGFBP-2, FGF-9, FLT3L GCP-2, and NT3 are key downregulated cytokines (Figure 1B). Moreover, the patterns of cytokine expression in the macrophages are similar within melanoma and non-melanoma groups but distinct between them (Figures 1C, D). Most of these cytokines modulated by the melanoma-derived EVs on the naïve macrophages represent the induction of TPM-associated pathways (Supplementary Table S2). Interestingly, IL1β, IL8, and MCP1 are directly involved in the crosstalk with adipocytes in deregulated metabolism (Bing, 2015). CXCL13 is known to be secreted by M2 macrophages to promote cancer cell metastasis (Xie et al., 2021). In addition, MCP1 (CCL2) and MIP1β (CCL4) are considered TPM’s biomarkers (De la Fuente López et al., 2018).

### 3.2 Systemic regulation of cytokines in metastatic melanoma

**FIGURE 2:**
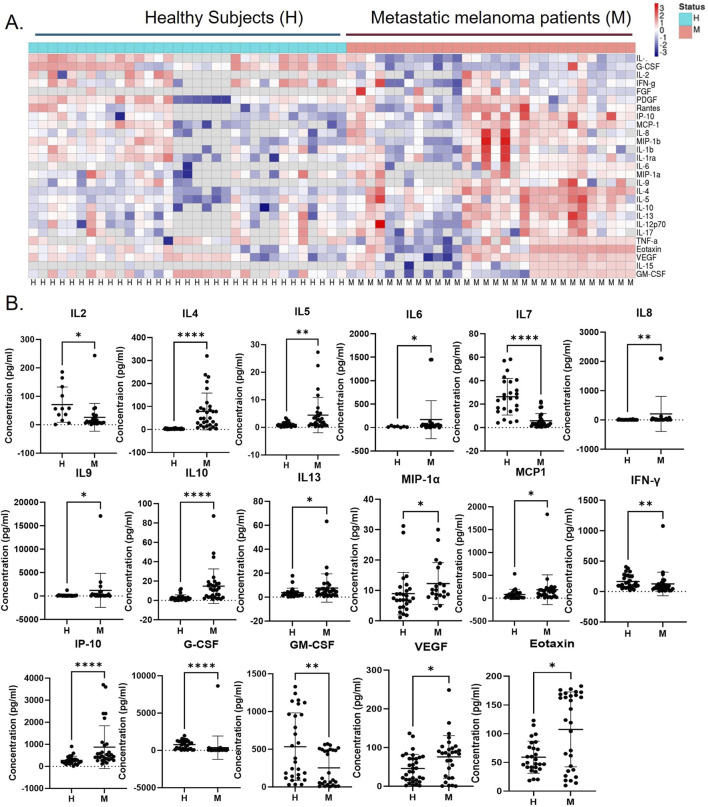
Immunosuppressive Th2 cytokine levels are upregulated in metastatic melanoma patients compared to healthy subjects. **(A)** The cytokine levels in the blood plasma of human stage IV metastatic melanoma (M; n = 30) and healthy subjects (H; n = 33) were analyzed using the Luminex kit, and the heatmap provided a summary of all analyzed cytokines, and **(B)** dot plots show cytokine levels between melanoma patients and healthy subjects with significant differences represented by *p ≤ 0.05, **p ≤ 0.01, ***p ≤ 0.001, and ****p ≤ 0.0001.

**FIGURE 3:**
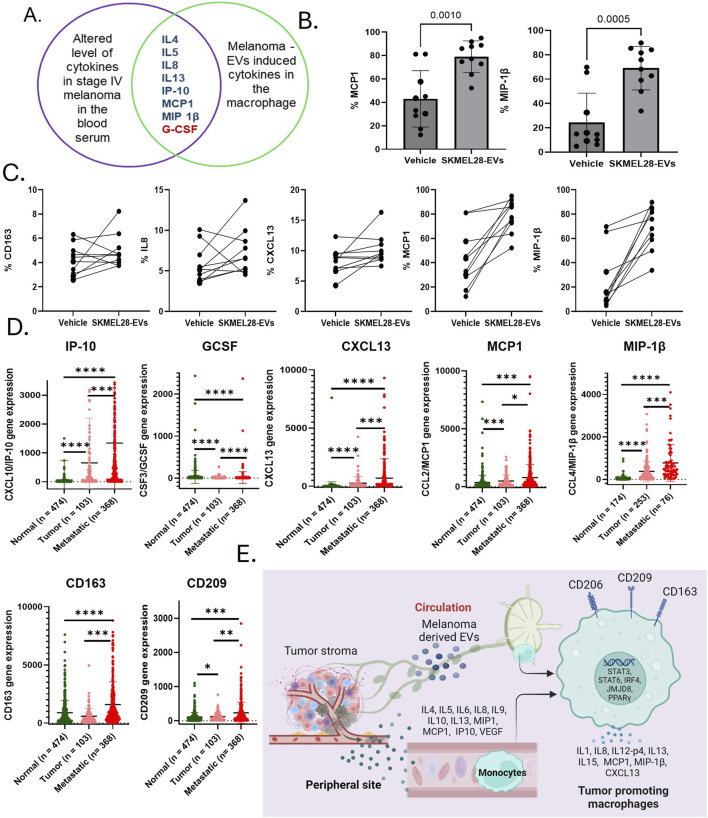
Immunosuppressive cytokines exhibiting tumor-promoting functions within macrophages have been implicated in dysfunction following exposure to melanoma-derived extracellular vesicles (EVs). **(A)** A Venn diagram shows the list of cytokines with similar regulation trends in the systemic and melanoma-derived EV-treated macrophages; **(B)** CCL2 and CCL4 are among the top upregulated cytokines by exposures of SKMEL28 EVs to human-derived macrophages (n = 10); **(C)** SKMEL28 EVs upregulate M2 macrophage markers CD163 and other tumor-promoting cytokines (IL-8, CXCL13, MCP1, and MIP1β) in naïve macrophages upon SKMEL28 EV treatment; **(D)** analysis of upregulated cytokines listed above in the Venn diagram in the SKCM dataset of normal tissue from non-cancer patients, tumor tissue, and metastatic melanoma tissue; and **(E)** data from this study summarize that melanoma-induced immunosuppression, driven by changes in the cytokine profile, plays a crucial role in the formation of the premetastatic niche and subsequent metastasis. This process is likely facilitated by the generation of tumor-promoting macrophages, which occurs through the modulation of various cytokines and transcriptional mechanisms after exposure to melanoma-secreted EVs.

The presence of systemic Th2 immune chronic inflammation in metastatic melanoma has been previously documented (Nevala et al., 2009). Our study was carried out to assess the levels of plasma cytokines in the metastatic stage of melanoma using the Luminex platform in newly diagnosed and previously untreated stage IV melanoma patients (n = 30) and healthy subjects (n = 33). The analysis identified 17 significantly altered cytokines, with 12 cytokines (IL4, IL5, IL6, IL8, IL9, IL10, IL13, MIP1, MCP1, IP-10, VEGF, and eotaxin) showing upregulation and five cytokines (IL2, IL7, IFN-γ, G-CSF, and GM-CSF) showing downregulation (Figure 2). Earlier research also revealed similar findings where melanoma patients associated with negative sentinel lymph nodes exhibit significantly elevated levels of IL-4, IL-6, and IL-10 compared to healthy controls, while IFN-gamma levels are notably lower (Porter et al., 2001). This suggests that a melanoma mediator including EVs may directly be involved in modulating these cytokines in systemic circulation. It is noteworthy that several cytokines with altered expressions in the melanoma patients’ blood (IL4, IL5, IL8, IL13, IP10, MCP1, MIP, and G-CSF) exhibited a similar expression level on melanoma EV-treated naïve macrophages (Figure 3A). Interestingly, all of these cytokines in macrophages are associated with the tumor-inflammatory environment and tumor-promoting functions (Chen et al., 2018). A previous study demonstrated that melanoma-secreted cytokines between primary and metastatic melanoma are significantly different (Elias et al., 2010). This finding highlights the role of melanoma cell behavior in promoting aggressiveness and mediating a tolerant immune microenvironment in the premetastatic niche. More importantly, melanoma cells directly target macrophages by releasing specific mediators in the form of EVs or cytokines to support the aggressive nature of metastatic melanoma cells (Wang et al., 2017a). This implies that melanoma-derived factors instigate systemic and local changes to establish an immunosuppressive environment through inducing tumor-promoting macrophages, which facilitate the metastasis of melanoma cells.

### 3.3 Tumor-promoting macrophages are key to provide immune tolerance through cytokine regulation and melanoma-derived EVs

TPMs have been implicated as a driver of tumor metastasis (Lin et al., 2019). Melanoma-derived EVs are important factors in the TME and contribute to the development of a niche that promotes tumor growth. The cytokine levels of IL4, IL5, IL8, IL13, IP10, MCP1, and MIP1β are commonly upregulated in the plasma of metastatic patients and melanoma EV-treated macrophages (Figure 3A). These cytokines are known for immunosuppressive macrophage activities that promote cancer progression (Supplementary Table S2). However, to know if melanoma-associated EVs transform macrophages, we further tested the key melanoma EV-induced macrophages cytokines and M2 macrophage surface markers in healthy PBMC-derived macrophages. For that, we treated M0 macrophages derived from healthy blood donors (n = 10) with SKMEL28 EVs. Interestingly, we found that MCP1 and MIP1β levels were upregulated in all the samples in the SKMEL28 EVs-treated group compared to the control group (Figures 3B, C). Moreover, IL-8 (8/10), CXCL-13 (9/10), and CD163 (8/10) levels were upregulated (Figure 3C), which validate that melanoma-derived EVs potentially transform macrophages to support tumor progression. Additionally, analysis of the TCGA-SKCM database revealed similar expression patterns of these gene sets in tumor and metastasis cases compared to the control, indicating the upregulation of tumor-promoting macrophages (CD163 and CD209) (Figure 3D). The heightened expression of cytokines in metastatic melanoma, either endogenously or resulting from induction in macrophages by melanoma-derived factors, signifies pivotal immunological mechanisms that have the potential to exacerbate the inflammatory microenvironment responses (Supplementary Figure S3). Several transcription regulators of TPMs also confirmed higher expression in metastatic melanoma patients in the TCGA-SKCM dataset (Supplementary Figure S4). Furthermore, a positive correlation between several immunosuppressive cytokines and TPM markers like CD163 was also observed in these datasets (Supplementary Figure S5). This evidence consistently supports the notion that factors secreted by tumors activate TPMs to modulate immune responses in melanoma as TPMs are pivotal in regulating T-lymphocytes and NK cells to facilitate metastasis (Yang et al., 2023). Notably, TPMs are recognized for their role in regulating a process akin to epithelial–mesenchymal transition, thereby promoting metastasis through the secretion of IL-8 (Fu et al., 2015). Cytokine array data show strong upregulation of IL-8, MCP1, CXCL13, and MIP1β among melanoma-derived EV-induced macrophage-associated cytokines, which signifies the role of these cytokines in inducing TPMs. Notably, MCP-1 is responsible for augmenting macrophage infiltration in the TME and acts as a potent macrophage-recruiting molecule, expressed in human malignant melanoma (Wang et al., 2017b). Moreover, MCP1 is positively correlated with the inflammatory activity of TPMs and enhances the tumor-promoting activation of the premetastatic systemic inflammatory T cell–IL17–neutrophil axis (Kersten et al., 2017). Strategies targeting TPMs and inhibiting MCP-1 have demonstrated efficacy in reducing angiogenesis and tumor growth in human melanoma xenografts (Gazzaniga et al., 2007). Previous studies also supported that MIP1β (Mukaida et al., 2020) and CXCL13 (Si and Hu, 2021; Chang et al., 2022) are involved in tumor promotion through their overexpression in macrophages.

In summary, our research findings demonstrate that melanoma-derived EVs can induce immunosuppressive activity in macrophages by upregulating levels of key cytokines, including IL-8, MCP-1, and CXCL13. Notably, our results revealed that these cytokine levels are significantly elevated in the blood plasma of patients diagnosed with metastatic melanoma, suggesting a systemic effect of the tumor on the immune environment. This also suggests that melanoma-associated cytokines and melanoma-derived EVs are interconnected with TPMs, highlighting their potential role in the progression of melanoma. The elevated levels of immunosuppressive cytokines (IL-4, IL-10, and IL-13) and decreased GM-CSF level in blood plasma of metastatic melanoma indicate their direct role in the transformation of macrophages into an immunosuppressive M2 phenotype (Hao et al., 2012). Our results strengthen that melanoma-derived EVs, which facilitate the communication between tumor cells and immune cells, ultimately create a favorable environment for tumor growth and metastasis by supporting the development of TPMs. Furthermore, the TCGA-SKCM dataset validates the transcriptional activation of immunosuppressive mechanisms through altered cytokine expression accountable for TPM generation within the TME, thereby bolstering metastasis.

## Data Availability

Publicly available TCGA-SKCM dataset was analyzed in this study. The data can be found on online analysis platforms at TNMplot.com and timer.cistrome.org.
