# Supplementary material for: Melanoma-derived cytokines and extracellular vesicles are interlinked with macrophage immunosuppression
Source: Front Mol Biosci. 2025 Jan 22;11:1522717. doi: 10.3389/fmolb.2024.1522717 (PMC11794111; doi:10.3389/fmolb.2024.1522717)
Supplement: Supplementary file 1 [file Presentation1.ppt]

## Slide 1
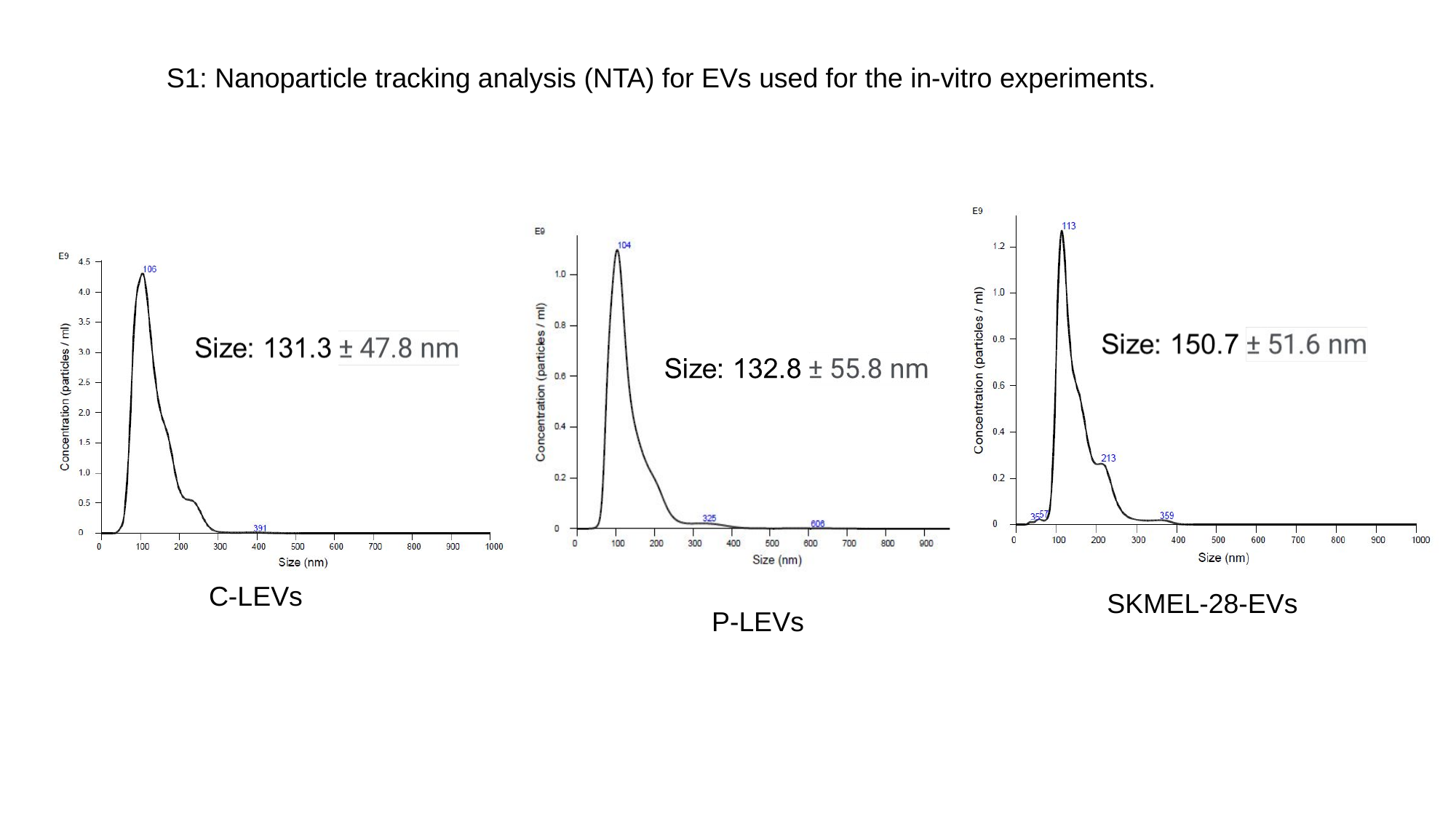

# S1: Nanoparticle tracking analysis (NTA) for EVs used for the in-vitro experiments.
C-LEVs
SKMEL-28-EVs
P-LEVs

## Slide 2
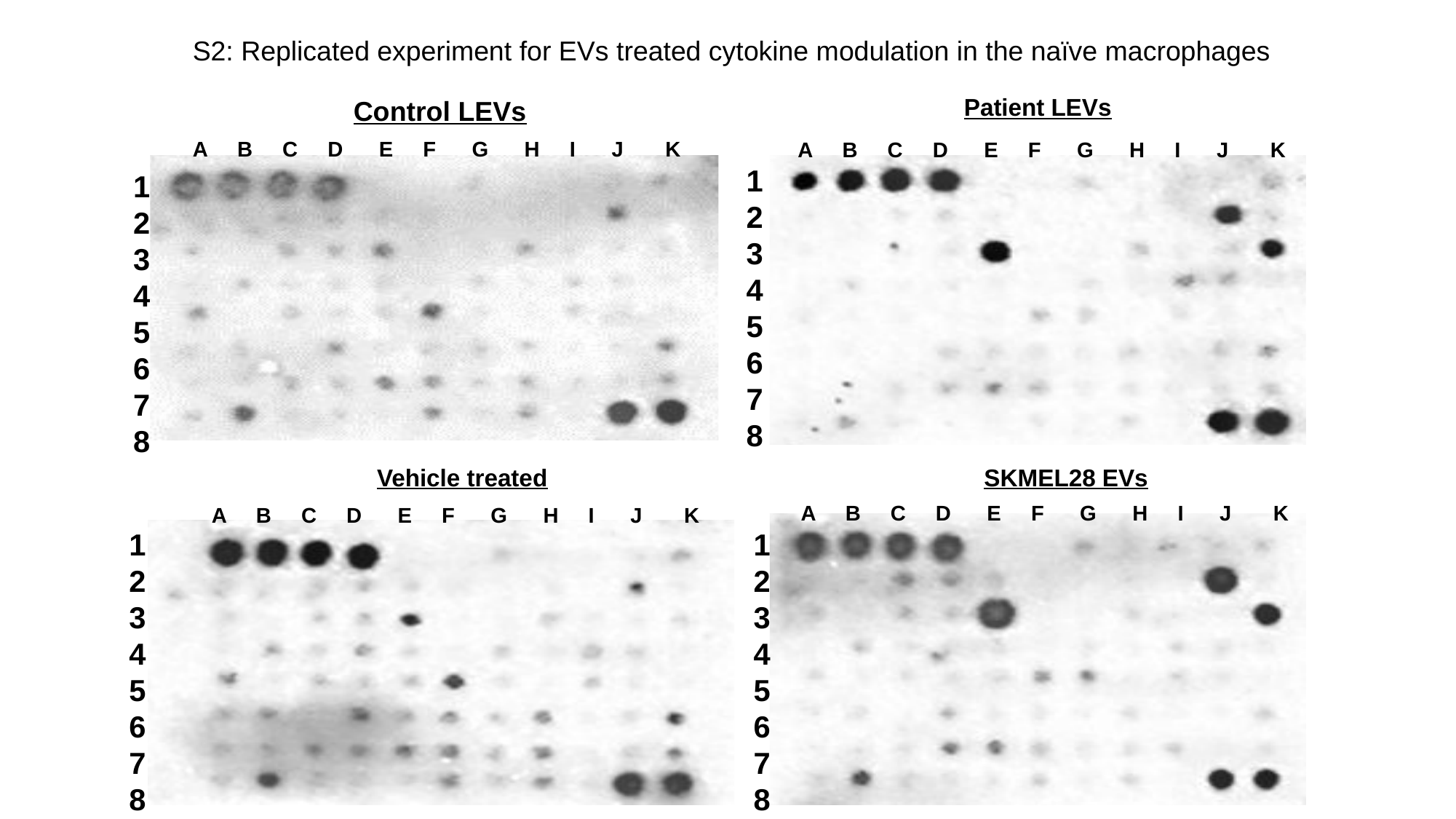

# S2: Replicated experiment for EVs treated cytokine modulation in the naïve macrophages
Patient LEVs
Control LEVs
A B C D E F G H I J K
A B C D E F G H I J K
1
2
345678
1
2
345678
SKMEL28 EVs
Vehicle treated
A B C D E F G H I J K
A B C D E F G H I J K
1
2
345678
1
2
345678

## Slide 3
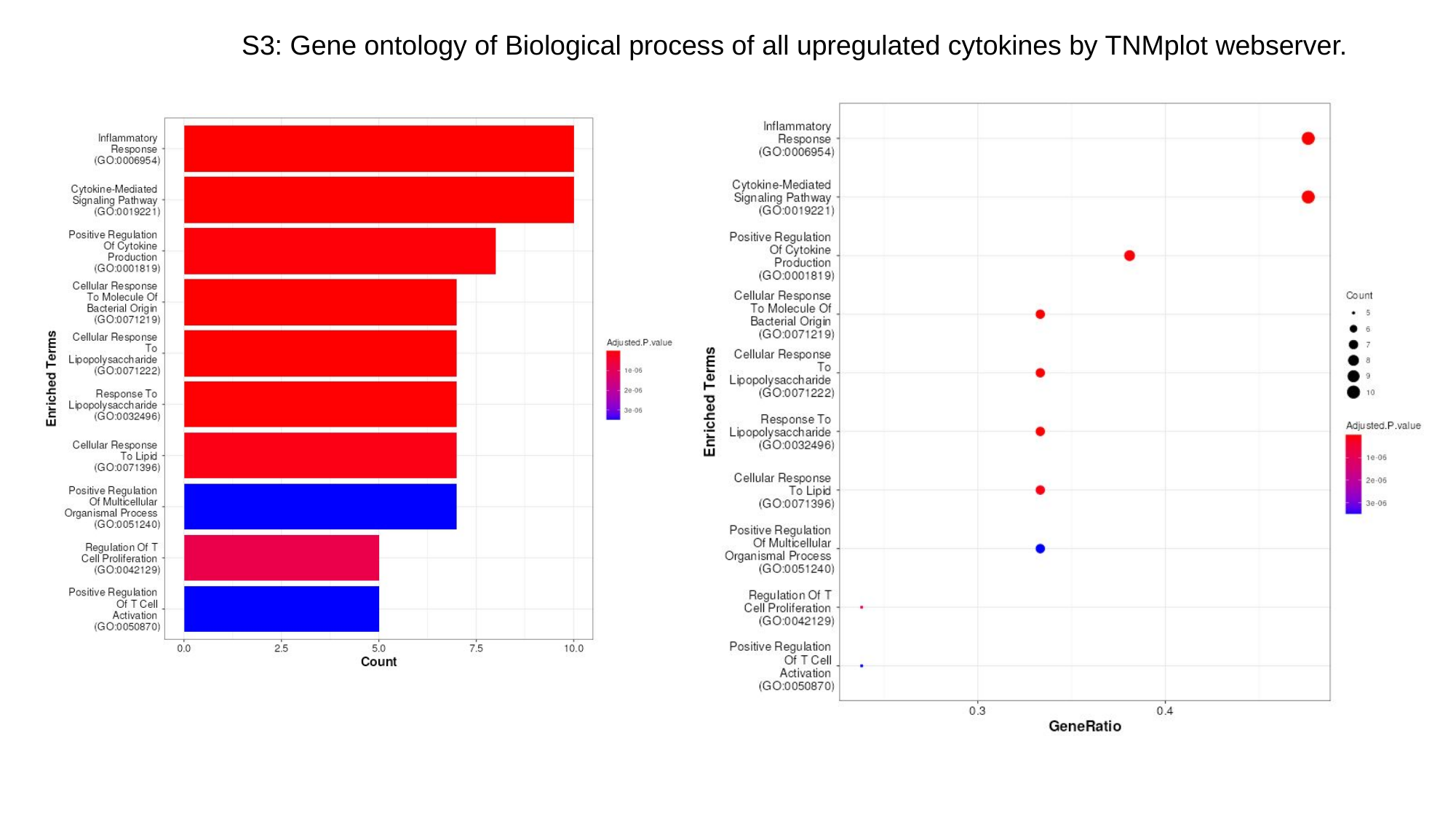

S3: Gene ontology of Biological process of all upregulated cytokines by TNMplot webserver.

## Slide 4
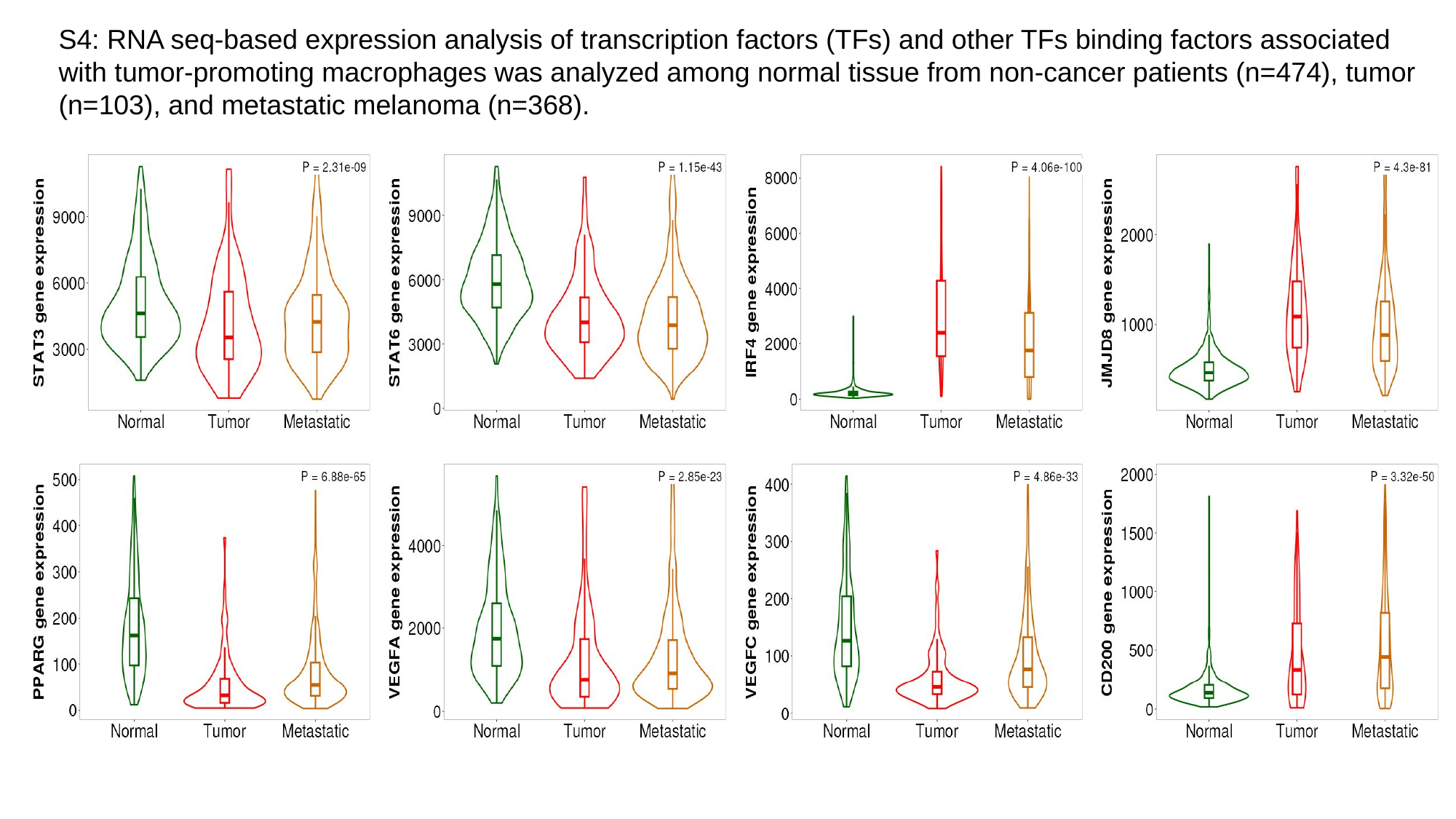

S4: RNA seq-based expression analysis of transcription factors (TFs) and other TFs binding factors associated with tumor-promoting macrophages was analyzed among normal tissue from non-cancer patients (n=474), tumor (n=103), and metastatic melanoma (n=368).

## Slide 5
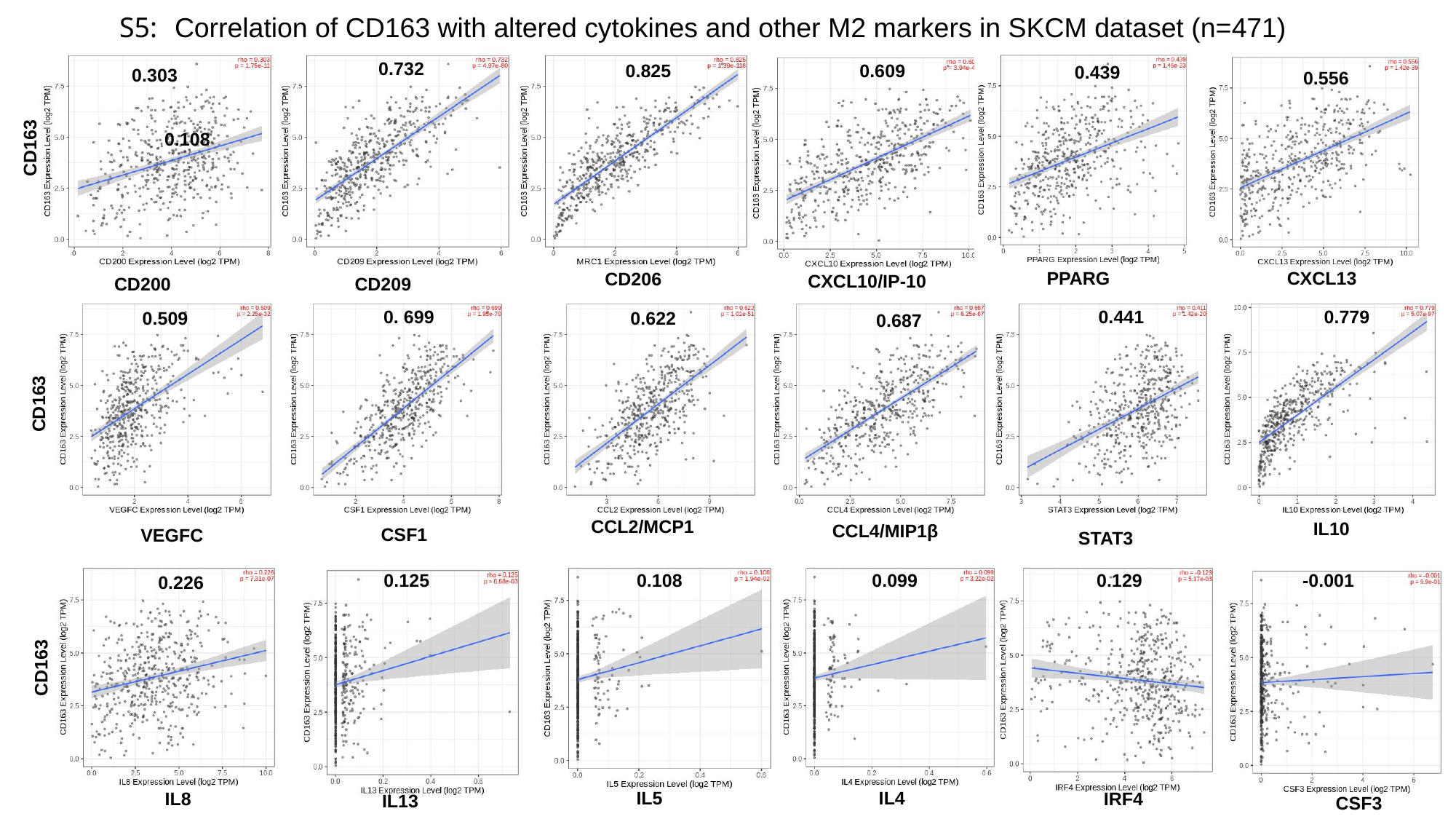

# Correlation of CD163 with altered cytokines and other M2 markers in SKCM dataset (n=471)
S5:
0.732
0.825
0.609
0.303
0.439
0.556
0.108
CD163
CXCL13
PPARG
CD206
CXCL10/IP-10
CD209
CD200
0.509
0. 699
0.622
0.687
0.441
0.779
CD163
CCL2/MCP1
IL10
CCL4/MIP1β
CSF1
VEGFC
STAT3
0.226
0.125
0.108
0.099
0.129
-0.001
CD163
IL5
IL4
IL8
IRF4
IL13
CSF3
