# Supplementary material for: Melanoma-derived cytokines and extracellular vesicles are interlinked with macrophage immunosuppression
Source: Front Mol Biosci. 2025 Jan 22;11:1522717. doi: 10.3389/fmolb.2024.1522717 (PMC11794111; doi:10.3389/fmolb.2024.1522717)
Supplement: Supplementary file 2 [file Table1.docx]

Table S1: Antibodies (Flow Cytometry)

| **Manufacturer** | **Catalog** | **Fluorophore** |
| --- | --- | --- |
| Biolegend | 511411 | IL8 (AF 488) |
| Biolegend | 502613 | CCL2/MCP1 PE-Cy7 |
| BD | 561120 | CCL4 PE , PE Mouse Anti-Human MIP-1β |
| Novus biologicals | IC8012U-100UG | Human CXCL13/BLC/BCA-1 Alexa Fluor® 350-conjugated Antibody |
| Biolegend | 333623 | PE/Dazzle594 Anti-Human CD163 Antibody |
| Biolegend | 321141 | Brilliant Violet 785 Anti-Human CD206 Antibody |
| Biolegend | 361607 | PerCP/Cyanine5.5 anti-human HLA-DR Antibody |

Table S2: Highlighted TAMs signatures possibly involve in the melanoma metastasis

| **Cytokines** | **Macrophages activity** |
| --- | --- |
| MCP1 | Migration and infiltration (Deshmane, Kremlev et al. 2009) |
| IL4, IL13 | M2 macrophage polarization(Little, Pathanjeli et al. 2019) |
| IL8, MIP1β,  CXCL13 | Tumor associated macrophages signalling(Ahmed, Mohamed et al. 2021)(Wierzbicki, Bednarz-Misa et al. 2024)(Chang, Chao et al. 2022) |
| IL5 | Induce Tregs(Tran, Hodgkinson et al. 2012) |
| IP-10 | Attract Treg cells (Lunardi, Lim et al. 2015) |

| **Receptors** | **Macrophages activity** | |
| --- | --- | --- |
| CD200 | increases alternatively activated macrophages(Hayakawa, Wang et al. 2016) | |
| CD206/MRC1 | Tumor Macrophages markers  (Scodeller, Simon-Gracia et al. 2017): | |
| CD209 | M2 Macrophage through Activation Markers (Roszer 2015) | |
| CD163 | A M2 macrophage markers, contribute to predict aggressiveness(Hu, Liu et al. 2017) | |
| **Transcription factors (TF) or TF binding factor** | **Macrophages activity** |  |
| Stat3 | M2 macrophage differentiation (Fu, Duan et al. 2017) |  |
| Stat6 | Promotes M2 Macrophage Polarization (Gong, Zhuo et al. 2017) |  |
| IRF4 | regulates M2 macrophage polarization (Satoh, Takeuchi et al. 2010) |  |
| JMJD8 | M2 Macrophage Biomarker involve in Immunosuppression in Pan-Cancer. (Liang, Zhang et al. 2022) |  |
| PPARG | primes human monocytes into alternative M2 macrophages (Bouhlel, Derudas et al. 2007) |  |
| CSF1 | Reprograms Macrophages to M2-Like(Chen, Lai et al. 2021). |  |

**References**

1. Ahmed, S., et al. (2021). "IL-8 secreted by tumor associated macrophages contribute to lapatinib resistance in HER2-positive locally advanced breast cancer via activation of Src/STAT3/ERK1/2-mediated EGFR signaling." Biochim Biophys Acta Mol Cell Res 1868(6): 118995.
2. Bouhlel, M. A., et al. (2007). "PPARgamma activation primes human monocytes into alternative M2 macrophages with anti-inflammatory properties." Cell Metab 6(2): 137-143.
3. Chang, S. J., et al. (2022). "The Diagnostic Significance of CXCL13 in M2 Tumor Immune Microenvironment of Human Astrocytoma." Pathol Oncol Res 28: 1610230.
4. Chen, Y. C., et al. (2021). "Withholding of M-CSF Supplement Reprograms Macrophages to M2-Like via Endogenous CSF-1 Activation." Int J Mol Sci 22(7).
5. Deshmane, S. L., et al. (2009). "Monocyte chemoattractant protein-1 (MCP-1): an overview." J Interferon Cytokine Res 29(6): 313-326.
6. Fu, X. L., et al. (2017). "Interleukin 6 induces M2 macrophage differentiation by STAT3 activation that correlates with gastric cancer progression." Cancer Immunol Immunother 66(12): 1597-1608.
7. Gong, M., et al. (2017). "STAT6 Upregulation Promotes M2 Macrophage Polarization to Suppress Atherosclerosis." Med Sci Monit Basic Res 23: 240-249.
8. Hayakawa, K., et al. (2016). "CD200 increases alternatively activated macrophages through cAMP-response element binding protein - C/EBP-beta signaling." J Neurochem 136(5): 900-906.
9. Hu, J. M., et al. (2017). "CD163 as a marker of M2 macrophage, contribute to predicte aggressiveness and prognosis of Kazakh esophageal squamous cell carcinoma." Oncotarget 8(13): 21526-21538.
10. Liang, X., et al. (2022). "JMJD8 Is an M2 Macrophage Biomarker, and It Associates With DNA Damage Repair to Facilitate Stemness Maintenance, Chemoresistance, and Immunosuppression in Pan-Cancer." Front Immunol 13: 875786.
11. Little, A. C., et al. (2019). "IL-4/IL-13 Stimulated Macrophages Enhance Breast Cancer Invasion Via Rho-GTPase Regulation of Synergistic VEGF/CCL-18 Signaling." Front Oncol 9: 456.
12. Lunardi, S., et al. (2015). "IP-10/CXCL10 attracts regulatory T cells: Implication for pancreatic cancer." Oncoimmunology 4(9): e1027473.
13. Roszer, T. (2015). "Understanding the Mysterious M2 Macrophage through Activation Markers and Effector Mechanisms." Mediators Inflamm 2015: 816460.
14. Satoh, T., et al. (2010). "The Jmjd3-Irf4 axis regulates M2 macrophage polarization and host responses against helminth infection." Nat Immunol 11(10): 936-944.
15. Scodeller, P., et al. (2017). "Precision Targeting of Tumor Macrophages with a CD206 Binding Peptide." Sci Rep 7(1): 14655.
16. Tran, G. T., et al. (2012). "IL-5 promotes induction of antigen-specific CD4+CD25+ T regulatory cells that suppress autoimmunity." Blood 119(19): 4441-4450.
17. Wierzbicki, J., et al. (2024). "Macrophage Inflammatory Proteins (MIPs) Contribute to Malignant Potential of Colorectal Polyps and Modulate Likelihood of Cancerization Associated with Standard Risk Factors." Int J Mol Sci 25(3).
